# Protective Effect of *Acacia nilotica* (L.) against Acetaminophen-Induced Hepatocellular Damage in Wistar Rats

**DOI:** 10.1155/2013/987692

**Published:** 2013-06-24

**Authors:** Narayanan Kannan, Kunnathur Murugesan Sakthivel, Chandrasekaran Guruvayoorappan

**Affiliations:** Department of Biotechnology, Karunya University, Karunya Nagar, Tamil Nadu, Coimbatore 641114, India

## Abstract

The potential biological functions of *A. nilotica* have long been described in traditional system of medicine. However, the protective effect of *A. nilotica* on acetaminophen-induced hepatotoxicity is still unknown. The present study attempted to investigate the protective effect of *A. nilotica* against acetaminophen-induced hepatic damage in Wistar rats. The biochemical liver functional tests Alanine transaminase (ALT), Aspartate transaminase (AST), Alkaline phosphatase (ALP), total bilirubin, total protein, oxidative stress test (Lipid peroxidation), antioxidant parameter glutathione (GSH), and histopathological changes were examined. Our results show that the pretreatment with *A. nilotica* (250 mg/kg·bw) orally revealed attenuation of serum activities of ALT, AST, ALP, liver weight, and total bilirubin levels that were enhanced by administration of acetaminophen. Further, pretreatment with extract elevated the total protein and GSH level and decreased the level of LPO. Histopathological analysis confirmed the alleviation of liver damage and reduced lesions caused by acetaminophen. The present study undoubtedly provides a proof that hepatoprotective action of *A. nilotica* extract may rely on its effect on reducing the oxidative stress in acetaminophen-induced hepatic damage in rat model.

## 1. Introduction

Liver disease is one of the major health problems worldwide because liver is a vital organ has a wide range of functions in the body, including biotransformation and detoxification of endogenous and exogenous harmful substances, plasma protein synthesis, and glycogen storage [1]. Hepatic injury is associated with distortion of various metabolic functions. It is well known that reactive oxygen and nitrogen species play a crucial role in initiation and progression of liver-associated diseases such as alcoholic and viral hepatitis, nonalcoholic steatosis, and hepatocellular carcinoma [2–4]. The progression of liver fibrosis may develop into cirrhosis and is associated with liver cancer. Nearly 10–20% of patients' progress to cirrhosis which further leads to increasing the risk of hepatocellular carcinoma [5]. Steroids, vaccines, and antiviral drugs have been employed for treatment of liver diseases which have adverse side effects if administrated for long term. Extensive studies reported that natural products with antioxidant activity are effective to prevent the oxidative stress-related liver pathologies due to particular interactions and synergisms [6].

Acetaminophen (paracetamol) is widely used as analgesic and antipyretic drug. Acetaminophen is primarily metabolized by the liver and excreted by the kidneys; it is safe and effective when we use lower dose of acetaminophen, but excessive usage of acetaminophen can damage liver and the toxicity is not only associated with drug but also from one of its metabolite N-acetyl-p-benzoquinone imine (NAPQI) which is conjugated by hepatic glutathione to yield a product called mercapturic acid. Due to overdose of paracetamol, the glucuronidation and sulfation capacity is exceeded with formation of excess NAPQI. Liver damage is associated with depletion of glutathione, at this condition excessive NAPQI will bind with hepatic cell proteins and causes liver injury [7–9]. 

Herbal products and traditional medicines with better effectiveness and fewer side effects in therapeutics have replaced the synthetically derived drugs in modern allopathic medication system [10]. Many bioactive compounds and extracts from plants such as *Rosa damascena*, *Boerhaavia diffusa, Moringa oleifera, *curcumin from* Curcuma longa*, meso-zeaxanthin, and Salidroside from *Rhodiola sachalinensis* have thus been investigated for hepatoprotective and antioxidant effects against hepatotoxin-induced liver damage [11–16]. Therefore, there is a great demand for development of an effective hepatoprotective drug from the natural products. Traditional healers of different regions in India used *Acacia* species for treatment of various ailments [17]. *Acacia* species is one of the richest resources of bioactive flavonoids, alkaloids, phenolics, saponins, polysaccharides, tannins, and terpenoids [18]. The published reports of various biological activities of *Acacia* species include hypoglycemic, anti-inflammatory, antitumor [19], antifungal [20], antiplatelet aggregation, spasmogenic and vasoconstrictor, antihypertensive, antihepatitis C virus [21], antioxidant potential [22], wound healing [23], antinociceptive activity [24], chemopreventive and antimutagenic [25], and anthelmintic activity [26].

Among the acacia species, *A. nilotica* Subsp. *indica* is widely distributed in tropical and subtropical countries belonging to family Mimosoideae. Several bioactive agents have been identified from* A. nilotica* which includes androstene steroid, gallic acid, ellagic acid, kaempferol, naringenin, rutin, lupane, niloticane, umbelliferone catechin, and *γ*-sitosterol [21, 27–29]. Recently, studies from our laboratory showed protective effect of *A. nilotica* against Dalton's Lymphoma Ascites-induced tumor models [30]. However, several biological activities of *A. nilotica* have been reported; there is no scientific evaluation available in support of the hepatoprotective activity of *A. nilotica*. Based on its diversified pharmacological properties and its uses in traditional Indian system of medicine, in this study, an attempt was made to study its hepatoprotective activity against the acetaminophen-induced hepatotoxicity in comparison with standard drugs Silymarin and Liv52 in rat model.

## 2. Materials and Methods

### 2.1. Collection of Plant Material

The fresh aerial parts of the* A. nilotica *plant were collected from Annur near Coimbatore, India. The plant was authenticated at Botanical Survey of India, Coimbatore (BSI/SRC/5/23/2012-13/tech-459). The voucher specimen of the plant has been retained in the Department of Biotechnology, Karunya University, Coimbatore. The collected plant sample was washed thoroughly with running tap water and completely shade dried under room temperature.

### 2.2. Preparation of Extract

The shade dried aerial parts of the plant were subjected to mechanical size reduction. Then, the powdered material was extracted with methanol by using soxhlet apparatus. The solvent was removed by evaporation and extract was concentrated by using vaccum rotatory evaporator. The yield of the extract was found to be 10.5%.

### 2.3. Animals

Male Wistar Albino rats of body weights ranging from 150 to 160 g were obtained from Animals Breeding Station, Mannuthy, Thrissur. The animals were fed with standard pellet diet (Sai Durga feeds, Bangalore, India) and water ad libitum. They were maintained in controlled environment (12:12 h light/dark cycle) and temperature (30 ± 2°C). All the animal experiments were performed according to the Guidelines of the Institutional Animal Ethical Committee, Govt. of India.

### 2.4. Drugs and Chemicals

Silymarin was purchased from Microlabs, Bangalore, India. Liv52, a polyherbal formulation, consisting of hepatoprotective herbs (*Capparis spinosa, Cichorium intybus, Solanum nigrum, Terminalia arjuna, Cassia occidentalis, Achillea millefolium,* and *Tamarix gallica*) used as hepatic stimulant [31] was purchased from Himalaya Drug Company Bangalore, India. Acetaminophen (500 mg) tablet was purchased from Cipla Ltd., India. Gum acacia was purchased from Hi-Media (Mumbai, India). AST, ALP, ALP, and total bilirubin standard kits were purchased from Span Diagnostics Surat, India. Thiobarbituric acid, and nitroblue tetrazolium (NBT) were purchased from Sigma Aldrich, India. All other chemicals used were analytical reagent grade. 

### 2.5. Toxicity Studies

Acute *in vivo* toxicity studies with different concentrations of *A. nilotica* methanolic extract were carried out to determine the LD_50_ value by the Miller and Tainter method [32]. No deaths or adverse effects were detected during the 24-hour observation period in mice treated with up to 3000 mg/kg·bw of *A. nilotica* extract. Based on these results and the previous literature reports, the dose at the concentration of 250 mg/kg·bw was chosen for the experiments [33–35].

### 2.6. Treatment Design

Animals (Male Wistar rats) were randomized and divided into five groups (I–V) of six animals each. Group I served as untreated control and fed orally with normal saline 1 mL with 1% gum acacia (Vehicle alone). Group II was treated with acetaminophen alone at a dose of 2 g/kg·bw. Groups III and IV were treated with standard drugs Silymarin at a dose of 50 mg/kg·bw and Liv52 (1 mL/rat/day) for 7 consecutive days. Group V was treated with *A. nilotica *methanolic extract at a dose of 250 mg/kg·bw resuspended with 1% gum acacia for 7 consecutive days. To determine the effect of *A. nilotica* extract, rats were pretreated orally with *A. nilotica* extract for 7 consecutive days before acetaminophen suspension. On day 8, single dose of acetaminophen suspension (2 g/kg·bw) was given orally to all groups (II–V) except Group I.

### 2.7. Assessment of Hepatoprotective Activity

#### 2.7.1. Biochemical Estimations

On day 9, after 24 hour of acetaminophen administration, blood samples were collected by direct cardiac puncture using light ether anesthesia. Serum was separated by centrifuging at 2500 rpm for 15 min and used for analysis of AST, ALT, ALP, and total bilirubin by using standard Kits (Span Diagnostics Surat, India). Total protein was measured by using Lowry et al. [36].

#### 2.7.2. Antioxidant Parameters

On day 9, after collection of blood samples, all the animals were sacrificed via cervical dislocation and the livers were removed and weighed immediately. The liver index was calculated according to the formula: (rats liver weight/rats weight × 100%) using Huang et al. [37]. Livers were washed with ice cold saline and a 10% homogenate was prepared in 0.05 M sodium phosphate buffer (pH. 7.0). Then, the homogenate was centrifuged at 700 ×g for 10 min at 4°C and the collected supernatant used for estimation of lipid peroxidation (LPO) and reduced glutathione (GSH) by using standard methods Ohkawa et al. [38] and Szasz et al. [39], respectively.

### 2.8. Histopathological Analysis

A small portion of liver was taken and fixed in 10% formaldehyde. After several treatments for dehydration in alcohol, sections having 4 *μ*m thickness were cut and stained with haematoxylin and eosin, and histopathological analysis was carried out for the treated as well as (acetaminophen alone) control group of mice. 

### 2.9. Statistical Analysis

Values are expressed as mean (±SD). The statistical analysis was performed using one-way analysis of variance (ANOVA) followed By Dunnett's test using Graphpad InStat version 3.0, GraphPad Software, San Diego, CA, USA. *P* values (i.e., *P < 0.05, **P < 0.01) were considered statistically significant when compared to (acetaminophen alone) control.

## 3. Results

### 3.1. Effects of *A. nilotica* Extract on AST, ALT, and ALP Levels

**Figure 1:**
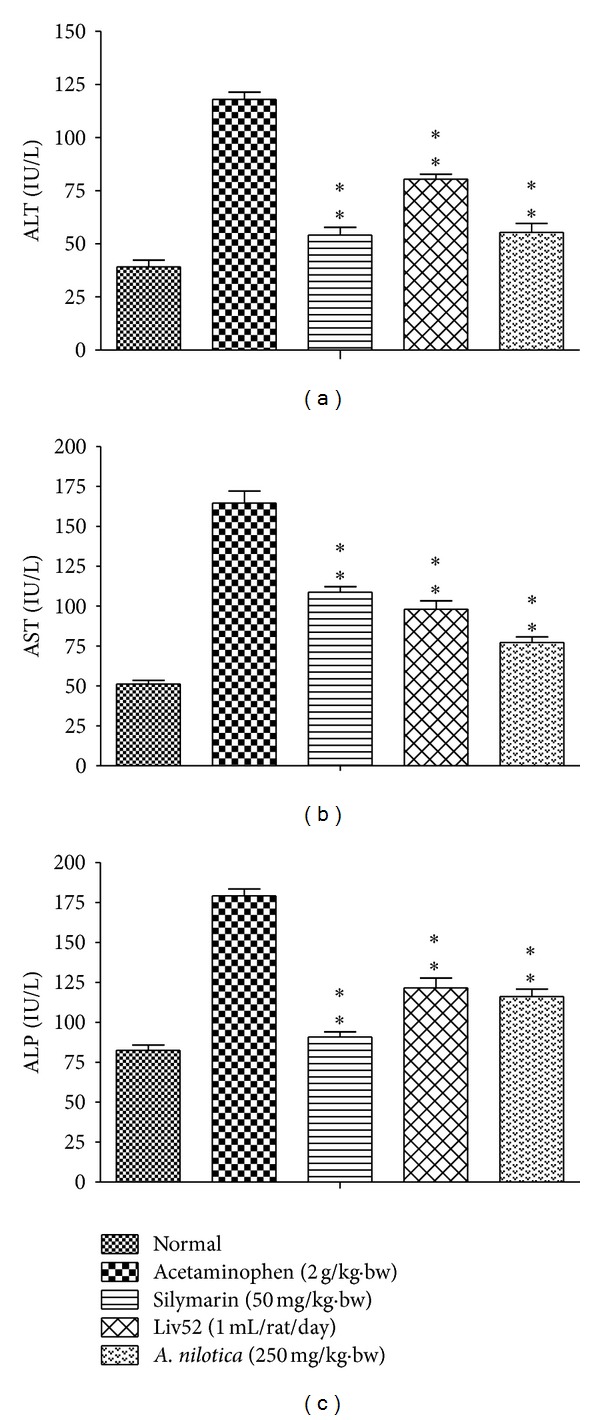
Effect of the methanolic extract of *A. nilotica* (250 mg/kg·bw) on ALT, AST, and ALP in acetaminophen-treated animals. Values shown are the means (±SD)—expressed in IU/L of 6 mice/treatment group. Values are significantly different from acetaminophen-treated (nonextract-treated) control, (*P < 0.05, **P < 0.01).

The effects of *A. nilotica *on serum AST, ALT, and ALP levels in acetaminophen-induced hepatotoxicity animals were represented in Figure 1. The levels of AST, ALT, and ALP in serum day 8 were significantly increased in acetaminophen alone treated control group (164.55 ± 7.53, 118.06 ± 3.39 and 179.26 ± 4.27 IU/L), respectively, as compared to the AST, ALT, and ALP of normal group (51.28 ± 2.37, 39.15 ± 3.14 and 82.57 ± 3.30 IU/L), respectively. After administration of *A. nilotica* methanolic extract to the acetaminophen-treated animals, the levels of AST, ALT, and ALP were significantly (**P < 0.01) reduced (77.33 ± 3.45, 55.41 ± 4.21 and 116.19 ± 4.72 IU/L), respectively, as compared to the acetaminophen alone treated animals (no treatment). Administration of standard drugs silymarin and Liv52 also significantly (**P < 0.01) reduced these levels (108.7 ± 3.60, 54.17 ± 3.63, 90.97 ± 3.12 IU/L, and 98.15 ± 5.25, 80.41 ± 2.38, 121.56 ± 6.22 IU/L), respectively, when compared with the *A. nilotica*-treated group in acetaminophen induced animals. 

### 3.2. Effects of *A. nilotica* Extract on Total Bilirubin and Total Protein Levels

**Figure 2:**
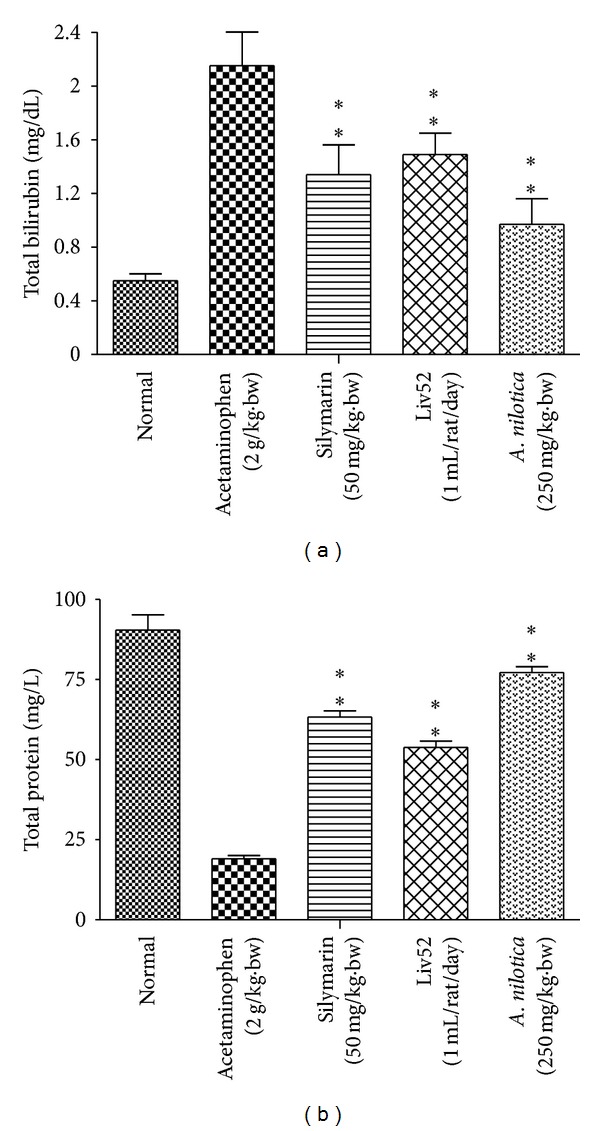
Effect of the methanolic extract of *A. nilotica* (250 mg/kg·bw) on total bilirubin (mg/dL) and total protein levels (mg/L) in acetaminophen-treated animals. Values shown are the means (±SD) of 6 mice/treatment group. Values are significantly different from acetaminophen-treated (nonextract-treated) control, (*P < 0.05, **P < 0.01).

The effects of *A. nilotica *on serum total Bilirubin and total protein levels were represented in Figure 2. The level of total bilirubin in serum on day 8 was significantly increased in acetaminophen alone treated control group (2.14 ± 0.25 mg/dL) compared to the total bilirubin of normal group (0.55 ± 0.05 mg/dL). After administration of *A. nilotica* methanolic extract to the acetaminophen-treated animals, the levels of total bilirubin were significantly (**P < 0.01) reduced (0.97 ± 0.19 mg/dL) when compared to the acetaminophen alone treated animals (no treatment). Administration of standard drugs silymarin and Liv52 also significantly (**P < 0.01) reduced the total bilirubin level (1.34 ± 0.22 and 1.49 ± 0.16 mg/dL), respectively, when compared with the *A. nilotica*-treated group in acetaminophen-induced animals. The level of total protein was significantly (**P < 0.01) decreased in acetaminophen-treated control group (19.1 ± 1.08 mg/L) when compare to normal control (90.46 ± 4.76 mg/L). Administration of *A. nilotica *extract significantly increased the total protein level up to (77.24 ± 1.84 mg/L), whereas standard drugs silymarin and Liv52 also increased the level up to (63.26 ± 1.98 and 53.79 ± 2.02 mg/L), respectively. 

### 3.3. Effects of *A. nilotica *Extract on Liver Weight, Liver Index, LPO, and GSH

**Figure 3:**
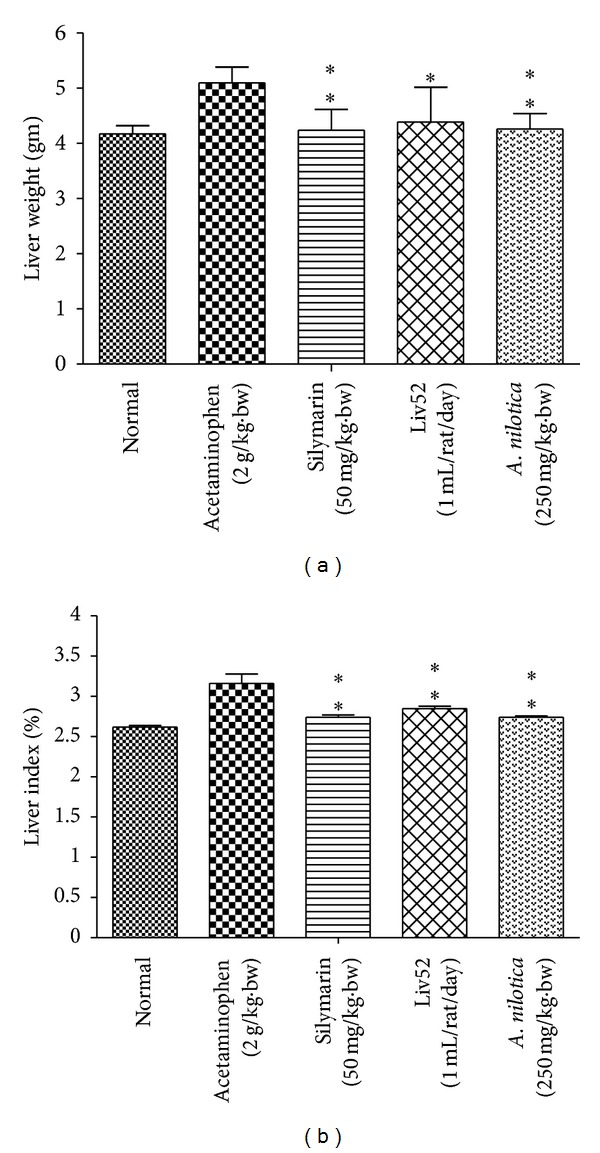
Effect of the methanolic extract of *A. nilotica* (250 mg/kg·bw) on liver weight (gm) and liver index (%) in acetaminophen-treated animals. Values are significantly different from acetaminophen treated (nonextract-treated) control, (*P < 0.05, **P < 0.01).

**Figure 4:**
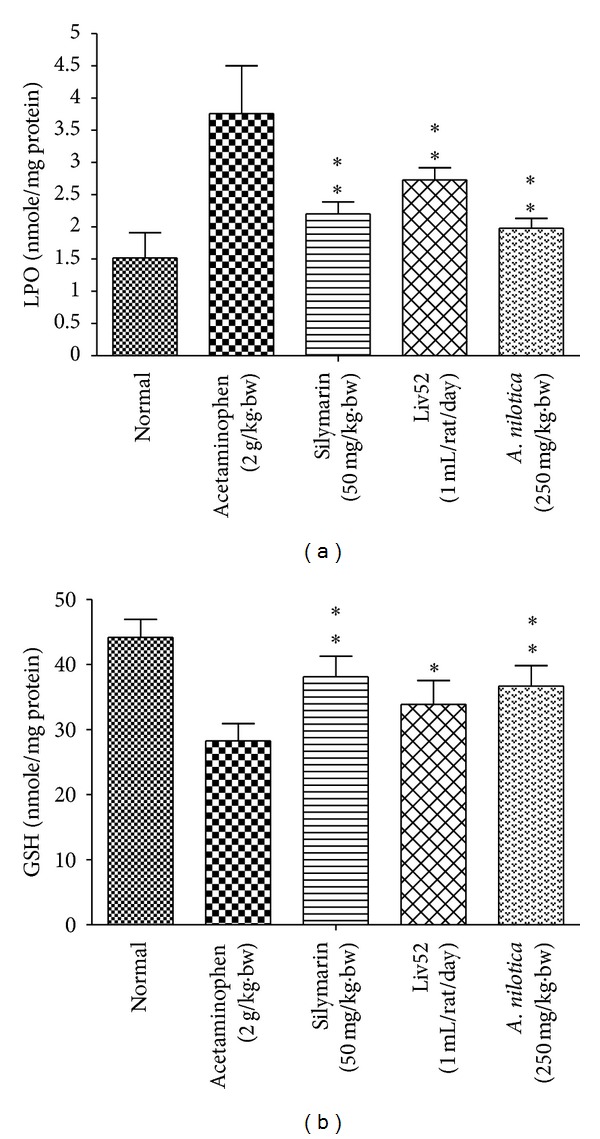
Effect of the methanolic extract of *A. nilotica* (250 mg/kg·bw) on LPO and GSH levels in acetaminophen-treated animals. Values are significantly different from acetaminophen-treated (nonextract-treated) control, (*P < 0.05, **P < 0.01).

The effect of *A. nilotica* on mean liver weight was significantly (**P < 0.01) reduced in acetaminophen-treated control group (4.26 ± 0.28 g) when compare to hepatotoxic control (5.10 ± 0.28 g), similarly the standard drugs silymarin and Liv52 were also decreased the mean liver weight up to (4.24 ± 0.38 and 4.39 ± 0.63 g), respectively. Furthermore, the liver index of acetaminophen-treated control group increased more markedly than that of *A. nilotica*, silymarin, and Liv52 treated animals (Figure 3). The effect of *A. nilotica* on the level of LPO (Figure 4) was significantly increased (3.76 ± 0.74 nmole/mg protein) when compare to normal group (1.51 ± 0.39 nmole/mg protein). Treatment with extract markedly decreased the level of LPO up to normal range (1.98 ± 0.15 nmole/mg protein). The effect of* A. nilotica* extract was comparable to standard drugs silymarin and Liv52 (2.20 ± 0.19 and 2.73 ± 0.19 nmole/mg protein), respectively. Acetaminophen treatment caused a significant decrease in the level of GSH (28.25 ± 2.68 nmole/mg protein) when compared with normal control group (44.20 ± 2.75 nmole/mg protein). Treatment with extract restored the decreased level of GSH up to (36.68 ± 3.18 nmole/mg protein) normal range. The standard drugs silymarin and Liv52 also caused significant increase in GSH level up to (38.10 ± 3.12 and 33.90 ± 3.65 nmole/mg protein), respectively.

### 3.4. Effect of *A. nilotica *Extract on Histopathology

**Figure 5:**
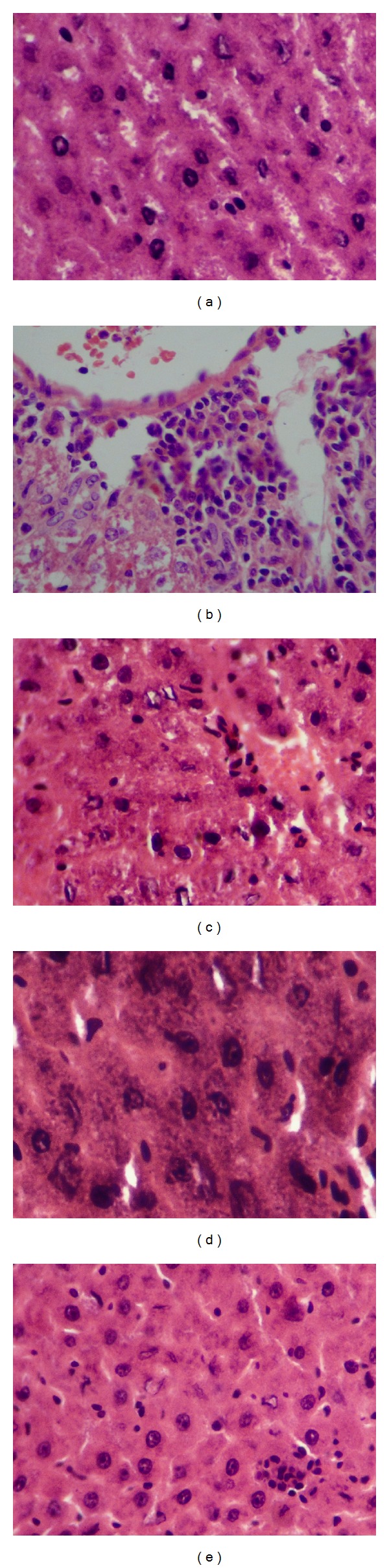
Histopathological changes in liver of experimental mice. Pictures shown are from representative liver sections collected at the end of the experimental period (a) normal; (b) acetaminophen-treated (nonextract treated) control; (c) acetaminophen + silymarin (d) acetaminophen + Liv52; (e) acetaminophen + *A. nilotica* extract.

Histopathological analysis revealed, normal untreated control group showed normal hepatocytes (Figure 5), and treatment with acetaminophen (no treatment) caused extensive vascular degenerative changes, sinusoidal dilation, central vein congestion, and central lobular necrosis. Treatment with *A. nilotica* extract produced mild degenerative changes and absence of necrosis, sinusoidal dilation, and central vein congestion. The treatment with standard drugs silymarin and Liv52 also showed normal hepatic architecture.

## 4. Discussion

Experiments were done to demonstrate the hepatoprotective potential of methanolic extract of *A. nilotica* in rat by acetaminophen-induced liver damage with pretreatment. Acetaminophen is a common antipyretic agent which is safe in therapeutic doses but can produce fatal hepatic necrosis in humans and animals with higher doses. Liver damage induced by the acetaminophen is a classical model for screening the hepatoprotective activity [40]. AST and ALT were found in serum and various body tissues but are mostly associated with liver parenchymal cells. The elevated level of AST and ALT will be observed in acute liver damage condition. In addition, the level of ALP will rise with intrahepatic cholestasis and infiltrative diseases of the liver [41]. The leakage of large quantities of enzymes into the blood stream was associated with centrilobular necrosis of the liver. Similarly in our study, increases in serum enzyme level of ALT, AST, and ALP after exposed to acetaminophen was observed and thereby confirms the hepatic structural damage. The levels of these enzyme levels have been restored up to normal range by *A. nilotica* treatment indicating its hepatoprotective action. The reliable criteria for judging the quality of any hepatoprotective drug are to preserve the normal hepatic physiological functions that have been disturbed by hepatotoxin [42]. Similar reports were observed from some other plants include *Aerva lanata, Plumbago* [43], and *Aegle marmelos *[44]. 

The levels of bilirubin and total protein in serum were related to the function of hepatic cell. A high concentration of bilirubin in serum is an indication for erythrocytes degradation rate caused due to liver injury when treated with hepatotoxin [45]. Diminution of total protein is a further indication of liver damage. The level of total protein will be decreased in hepatotoxic condition due to defective protein biosynthesis in liver [46]. In our study, the level of bilirubin and total protein has been restored towards the normal value indicating its hepatoprotective action. High dose of acetaminophen and its metabolite NAPQI can alkylate and oxidize the intracellular GSH and protein thiol group, which leads to GSH depletion and subsequently results in enhanced lipid peroxidation which leads to damage of liver [47]. Generally, our body has an effective defense mechanism to neutralize or prevent the toxicity produced by free radicals by using endogenous enzymes such as SOD and catalase. In acetaminophen-induced hepatotoxicity, the balance between ROS generation and antioxidant defense mechanism may be lost [48], thereby results in Oxidative stress, which leads to hepatic necrosis. Regarding nonenzymatic antioxidants, GSH which reduces hydrogen peroxide and xenobiotic toxicity acts as critical determinant of tissue susceptible to oxidative stress [49]. Depletion of GSH has been shown to be associated with enhanced toxicity to chemicals, including CCL_4_ and acetaminophen [50]. The results of our present study showed that the level of GSH was dramatically decreased in rats administrated with acetaminophen. However, pretreatments with *A. nilotica* and standard drugs silymarin and Liv52 markedly increased the level of GSH in acetaminophen-treated animals. 

MDA (the level of lipid peroxide) appears during peroxidation of biological membrane polyunsaturated fatty acids. The estimation of level of MDA is a measure of alterations and damage in structure of cellular membranes [51]. In our present study, increased levels of MDA in liver treated with acetaminophen suggest enhanced lipid peroxidation leading to tissue damage and failure to prevent formation of excess free radicals. Treatment with *A. nilotica* extract significantly reversed these changes, whereas similar results were also obtained in treatment with standard drugs. These results collectively suggest that the imbalanced antioxidant system in liver treated with acetaminophen is normalized by the protective effect of *A. nilotica* extract. The hepatoprotective effect of the *A. nilotica* extract was further accomplished by histopathological analysis. Histopathological findings of liver samples were in agreement with the results obtained in biochemical studies, indicating that *A. nilotica *extract is able to inhibit acetaminophen-induced hepatotoxicity. 

Phenolics and flavonoids display a wide range of biological and pharmacological properties and normally scavenge the free radicals and play an essential role in preventing oxidative stress. It is well documented that *A. nilotica* is one of the rich sources of these flavonoids and phenolics. For example, the polyphenolic compound Kaempferol displayed radical scavenging activity in different *in vitro* assays [52]. Niloticane isolated from the bark of the *A. nilotica* showed anti-inflammatory property by inhibition of cyclooxygenase enzymes which are involved in inflammatory process [28]. Similarly, gallic acid and catechin showed protective effect against N-nitrosodiethylamine-induced hepatocarcinogenesis [53]. Umbelliferone is also reported as potential scavenger of free radicals which is present in bark and leaves of *A. nilotica* [22].

The outcome of the present investigation undoubtedly indicates that the treatment with* A. nilotica* was effective on inhibiting the hepatotoxicity induced by acetaminophen in *in vivo* models, most likely because of high content of flavonoids, alkaloids, phenolics, steroids, terpenoids, saponins, and tannins and may be due to synergistic action of specific constituents present in the extracts such as umbelliferone, gallic acid, niloticane, and kaempferol derivatives may exert these preventing effects. However, the precise molecular mechanism by which *A. nilotica* mediates its hepatoprotective action is still not clear. Furthermore, we planned to identify more precisely the lead component responsible for hepatoprotective activity and to unveil the molecular mechanism behind its therapeutic action.
